# Plasma citrulline in the critically ill: intriguing biomarker, cautious interpretation

**DOI:** 10.1186/s13054-015-0881-1

**Published:** 2015-05-01

**Authors:** Gaël Piton, Gilles Capellier

**Affiliations:** Medical Intensive Care Unit, University Hospital, Besançon, 25030 France; Research Unit EA 3920 and SFR FED 4234, University of Franche Comté, Besançon, 25030 France; Department of Epidemiology and Preventive Medicine, School of Public Health and Preventive Medicine, Faculty of Medicine, Nursing and Health Sciences, Clayton, VIC 3168 Australia

## Abstract

In a recent issue of *Critical Care*, Poole and colleagues found that plasma citrulline concentration and glucose absorption were reduced in 20 critically ill patients compared with 15 controls; however, the authors found no correlation between these two variables. This study highlights the question of the accuracy of plasma citrulline for assessing small bowel function in critically ill patients. Future studies should take into account the type of intestinal failure considered, the particular metabolism of citrulline, the time of plasma citrulline measurement, as well as the range of citrullinemia considered.

In a recent issue of *Critical Care*, Poole and colleagues [1] evaluated the link between plasma citrulline concentration and glucose absorption in critically ill patients. This is an important contribution to the study of acute intestinal failure in the ICU.

Evaluating small bowel function and integrity is challenging in the critically ill [2]. Small bowel damage might be central in the development of systemic inflammatory response syndrome, bacterial translocation, and multiple organ failure [3]. In addition, small bowel damage might make it difficult or impossible to use the enteral route for feeding or medication intake. Citrulline is an amino acid not incorporated into proteins that is mainly synthesized by small bowel enterocytes [4]. Fifteen years ago, Crenn and colleagues [5,6] demonstrated that plasma citrulline concentration was a precise biomarker of small bowel function in patients with short bowel syndrome and villous atrophy-associated small bowel diseases, reflecting small bowel length and villi size, respectively. To date, numerous studies have confirmed these results in non-critically ill patients.

The question is to know whether plasma citrulline is also a valid biomarker in critically ill patients [7]. The paper of Poole and colleagues [1] is therefore welcomed. They confirmed that plasma citrulline concentration and glucose absorption were lower in 20 critically ill patients compared with 15 controls. Despite a trend toward a positive correlation (R = 0.28, *P* = 0.12), the link between plasma citrulline concentration and glucose absorption was not statistically significant. The authors concluded that plasma citrulline concentration was not a biomarker of glucose absorption in critically ill patients. As noted by the authors, however, the study was possibly underpowered to explore the link between plasma citrulline concentration and glucose absorption.

All in all, this apparently disappointing study highlights the need to clarify the interest and limits of plasma citrulline concentration in the critically ill. On the one hand, it is reasonable to assume that plasma citrulline concentration is related to small bowel function in the critically ill. First, the plasma citrulline concentration is low in more than half of critically ill patients [1,8,9]. Second, low plasma citrulline concentrations are associated with a poor prognosis [8,9]. Third, plasma citrulline and C-reactive protein concentrations are inversely correlated [8-10], and low plasma citrulline is associated with bacterial translocation [10,11]. Fourth, low plasma citrulline concentrations were found to be associated with elevated intestinal fatty acid binding protein concentration, a specific biomarker of enterocyte damage [11,12]. Fifth, plasma citrulline concentration was found to be lower among critically ill patients presenting with signs of intestinal dysfunction, such as feeding intolerance, ileus, diarrhea, or gastrointestinal bleeding [13]. On the other hand, plasma citrulline concentration might lack accuracy among critically ill patients. First, the metabolism of citrulline is complex. It depends on glutamine availability, its main precursor, on kidney function for the transformation of citrulline into arginine, on the level of systemic inflammation because nitric oxide synthase transforms arginine into nitric oxide plus citrulline, and on arginine bioavailability because citrulline might be overmetabolized as a source of arginine [4,14]. Second, the time of plasma citrulline measurement might be crucial. Indeed, plasma citrulline concentration describes a U-curve during ICU stay [8]. Even if plasma citrulline concentration is often low at the time of ICU admission, it is even lower 1 or 2 days later, whereas it tends to increase in survivors after 1 week. Because of this intra-individual variability, the time of measurement should be taken into account. Third, the present study suggests that plasma citrulline does not reflect correctly the absorptive function in the critically ill. Only few and preliminary studies have evaluated the links between plasma citrulline concentration, absorptive function, and barrier function in the ICU. Fourth, the range of normality for plasma citrulline concentration, which is between 20 and 40 μmol.L^−1^ in the stable patient, might not be adapted in the critically ill. Whereas a very low plasma citrulline concentration, ≤10 μmol.L^−1^, is likely to indicate an altered small bowel function, a plasma citrulline concentration between 10 and 20 μmol.L^−1^ might be in a grey zone for the interpretation [15].

In conclusion, we need additional data in this field. Due to its metabolism and particular kinetics in the critically ill, plasma citrulline concentration might not be as accurate as in stable patients for determining small bowel function. On the other hand, plasma citrulline is probably an indicator of small bowel function that should be integrated into a clinical context.
